# Supplementary material for: MetaRibo-Seq measures translation in microbiomes
Source: Nat Commun. 2020 Jun 29;11:3268. doi: 10.1038/s41467-020-17081-z (PMC7324362; doi:10.1038/s41467-020-17081-z)
Supplement: Supplementary file 10 — Supplementary Data 7 [file 41467_2020_17081_MOESM10_ESM.zip › File2/Confidence_High_Taxonomy/134589_out.krona.html]

Javascript must be enabled to view this page.

members
magnitude
magnitudeUnassigned
count
unassigned
taxon
rank

134589\_out

2

superkingdom
2

SRS014235\_contig\_number\_contig-100\_39845.39845
1
2

1239
1
phylum

class
1
186801

186802
1
order

186806
1
family

1
1730
genus

species
1
142586

SRS019068\_contig\_number\_39971
